# Treatment of mild subclinical hypothyroidism and its impact on lipid metabolism

**DOI:** 10.3389/fendo.2026.1847857

**Published:** 2026-06-30

**Authors:** Rachel Zielinski, Shane J. Sacco, Martha Dillon, Maria Katsetos, Francesco S. Celi

**Affiliations:** Department of Medicine, UConn Health, Farmington, CT, United States

**Keywords:** cholesterol, guidelines, hypothyroidism, levothyroxine, lipid metabolism, subclinical hypothyroidism

## Abstract

**Background:**

Hypothyroidism, both overt and subclinical, is associated with dyslipidemia and increased cardiovascular risk which is not completely corrected by thyroid hormone replacement therapy. In patients with subclinical hypothyroidism, when TSH levels are <10 mIU/L, dyslipidemia is considered a relative indication for establishment of replacement therapy. Although reasonable, the evidence supporting this approach is limited, and the results are conflicting. Here we present the results of a retrospective study directed to assess the differences in lipid metabolism parameters between patients with subclinical hypothyroidism treated or untreated with levothyroxine.

**Methods:**

Single-center, retrospective cohort study. Patients with diagnosis of mild subclinical hypothyroidism not taking thyroid hormone replacement therapy (at baseline) and at least two measurements of TSH and lipid panel were included in the analysis. Patients taking lipid lowering therapies were excluded from the study. Primary endpoints were changes in TSH, total and LDL cholesterol between patients treated and untreated with thyroid hormone replacement therapy.

**Results:**

Fifty-two patients (14 treated and 38 untreated) median age 61.0 years and body mass index 27.0 kg/m^2^ were included in the analysis. Median baseline TSH and LDL-cholesterol were 5.6 mIU/L and 121.0 mg/dL, respectively. At follow up, median TSH decreased in both groups (treated -2.5 mIU/L [95% CI: -3.8, -1.2] untreated -1.7 mIU/L [-2.6, -0.8] p<0.001). Median total and LDL-cholesterol significantly increased in the treated group (7.8 mg/dL [2.2, 13.5] p=0.01 and 13.2 mg/dL [1.4, 25.1] p=0.03, respectively), while the untreated group saw no changes. No changes were observed in HDL-cholesterol and triglycerides for either group.

**Conclusions:**

Our results indicate that in patients affected by mild subclinical hypothyroidism not receiving lipid lowering treatments, levothyroxine therapy was associated with increase in total cholesterol and LDL-cholesterol, while this did not occur in untreated patients. One possible explanation of this counterintuitive finding is the inhibition of endogenous thyroid hormone production with further reduction of T3-mediated metabolic on lipid metabolism. Prospective interventional studies are needed to confirm this observation, potentially changing the approach to the treatment of subclinical hypothyroidism.

## Introduction

Hypothyroidism is associated with dyslipidemia and increased cardiovascular risk (1), which is not completely reverted by levothyroxine (LT4) therapy (2). Subclinical hypothyroidism (SH), defined as an elevated thyroid-stimulating hormone (TSH) concentration above the upper limit of normal range and thyroid hormone concentrations within normal range is common in the general population (4-15%), and a risk factor for progression to overt hypothyroidism (2-5% per year) (3, 4). Both SH and overt hypothyroidism are associated with hypercholesterolemia and increased cardiovascular risk (5–7).

While the American Thyroid Association guidelines recommend treating SH when the TSH is ≥10 mIU/L (8), there is no consensus for when to treat SH patients with TSH <10 mIU/L (i.e., mild SH) (8). Because of the effects of thyroid hormone on lipid metabolism (1), hypercholesterolemia is considered a relative indication for LT4 treatment in patients affected by mild SH (9), but the evidence supporting this approach is limited and meta-analyses offer contrasting conclusions (10, 11).

Since the pituitary is particularly sensitive to T4, LT4 treatment of SH, which has thyroid hormones within normal range, might result in normalization of TSH at the cost of suppression of the production of endogenous T4 and T3 with decreased hormonal signaling to other tissues. Indeed, some studies suggest that LT4 therapy can result in a paradoxical decrease in T3 levels due to suppression of endogenous thyroid hormone production, leading to a *de facto* worsening of the hypothyroid state (12, 13). Given the prevalence of SH and the clinical significance of cardiovascular risk factors, there is an unmet need to better define the relationship between mild SH, dyslipidemia, and effects of LT4 therapy.

Here we present the results of a retrospective study directed to explore the effects of LT4 therapy in a cohort of patients affected by mild SH not treated with lipid lowering drugs.

## Methods

### Study design and participants

This was a single-center retrospective cohort study of mild SH patients attending outpatient clinics at the University of Connecticut Health Center identified between March 2021 and March 2024. Patients with primary diagnosis of SH and at least two TSH and two lipid profiles determinations were included in the analysis. Endpoints of the study were changes in TSH and in lipid profile parameters from baseline, after stratification of the study population according to LT4 therapy. Records were individually assessed by members of the research team (RZ, MD). Demographics, treatment, TSH, and lipid panel data were extracted from the electronic health platform (Epic). Patients who had a normal-range free T4 (defined by the laboratory reference range) and a TSH between 4.5 and 10.0 mIU/L not taking LT4 therapy at baseline (defined as the oldest available determination of TSH) were included in the analysis. Patients who were under 18 years, did not have a follow-up TSH or lipid panel, were treated for malignancy, had history of Graves’ disease or toxic multinodular goiter, were terminally ill or pregnant, or on medications which could affect thyroid metabolism (*e.g.* T3 supplementations, liothyronine, desiccated thyroid extracts, amiodarone, lithium) (14), or serum lipids (HMG-CoA reductase, fibrates or PCSK9 inhibitors), or receiving any chemotherapy regimen were excluded from the analysis. The UConn Health Institutional Review Board approved this study, and a waiver of informed consent was obtained prior to beginning the data collection.

### Statistical analysis

Study variables were described using frequencies and proportions for categorical variables and medians and interquartile ranges for numerical variables. Cohort characteristics were first compared by LT4 treatment via chi-squared tests and Wilcoxon ranked sum tests where appropriate. To more thoroughly compare changes over time in TSH and lipid measures within and between treatment groups, quantile regression models were employed. To adjust for baseline differences between treatment groups, the following variables were included in the model: age, sex, White race, and body mass index; baseline measure, and duration between baseline and follow-up. Outlying baseline lipid values were removed before regression analyses. For statistically significant group comparisons, and where appropriate, we provide the effect sizes Phi (φ) or absolute Cliff’s Delta (δ), which range from 0 (no effect) to 1 (maximum effect). Alpha was set to 0.05. Analyses were conducted in R 4.5.1 (15).

## Results

In **Table 1**, we summarize cohort characteristics as stratified by LT4 treatment. Our cohort included 53 patients: 14 treated patients (age range=28.0-83.0 years; LT-4 dose median=50.0 mcg; range=25.0-125.0) and 38 untreated patients (age range=24.0-88.0 years). Descriptively, treated patients appeared to have a lower median age (52.5 vs. 63.5 years), were more likely female (78.6% vs. 63.2%) and less likely Non-Hispanic White (57.1% vs. 71.1%). Treated patients also appeared to have lower median lipid levels at baseline (e.g., LDL-cholesterol: 112.0 vs. 121.5 mg/dL or 2.9 vs. 3.2 mmol/L) while having higher triglyceride levels (128.0 vs. 117.0 mg/dL or 1.5 vs. 1.3 mmol/L). However, none of these differences reached statistical significance.

**Table 1:** Cohort characteristics stratified by LT4 treatment.

LT4 treated			
Variable	Yes	No	p-value
No of patients, n (%)	14 (26.9)	38 (73.1)	
Age, y, median (Q_1_, Q_3_)	52.5 (42.0, 62.5)	63.5 (48.5, 72.0)	0.20
Biological sex, n (%)			0.34
Female	11 (78.6)	24 (63.2)	
Male	3 (21.4)	14 (36.8)	
Race and ethnicity, n (%)			0.51
Non-Hispanic White	8 (57.1)	27 (71.1)	
Non-Hispanic Black	1 (7.1)	1 (2.6)	
Hispanic or Latine	2 (14.3)	2 (5.3)	
Non-Hispanic Other or unknown	3 (21.4)	8 (21.1)	
Body mass index, kg/m^2^, median (Q_1_, Q_3_)	28.4 (25.5, 31.5)	25.6 (22.3, 31.7)	0.26
TSH, mIU/L, median (Q_1_, Q_3_)			
Baseline	6.0 (5.4, 7.1)	5.5 (5.1, 6.5)	0.28
Follow-up	2.4 (1.3, 4.3)	4.4 (3.2, 6.0)	0.01
Within-patient change	-3.5 (-4.7, -1.0)	-1.2 (-2.5, -0.2)	0.03
CHL, mg/dL, median (Q_1_, Q_3_)			
Total			
Baseline	197.0 (173.0, 218.8)	207.5 (182.5, 226.0)	0.61
Follow-up	202.0 (183.0, 214.8)	193.5 (166.2, 223.2)	0.39
Change	7.5 (-10.2, 23.8)	-7.0 (-24.0, 8.8)	0.05
LDL			
Baseline	112.0 (100.2, 135.2)	121.5 (104.8, 135.5)	0.48
Follow-up	123.5 (115.5, 135.5)	115.0 (93.2, 132.0)	0.18
Change	12.5 (-3.0, 23.5)	-4.5 (-20.5, 6.5)	0.02
HDL			
Baseline	48.0 (43.2, 51.5)	54.0 (46.0, 62.5)	0.11
Follow-up	49.5 (42.2, 55.8)	53.5 (46.0, 63.5)	0.28
Change	2.0 (-1.8, 7.8)	2.5 (-4.0, 6.8)	0.53
Triglycerides, mg/dL, median (Q_1_, Q_3_)			
Baseline	128.0 (93.0, 182.5)	117.0 (87.8, 153.0)	0.42
Follow-up	122.0 (89.8, 160.8)	103.5 (82.2, 153.0)	0.43
Change	-13.5 (-37.0, 33.8)	-18.0 (-36.5, 24.5)	0.91

TSH, Thyroid-stimulating hormone. CHL, Cholesterol. LDL, LDL cholesterol. HDL, HDL cholesterol. Conversion CHL, LDL, HDL mg/dL to mmol/L multiply by 0.0259. Conversion. *triglycerides mg/dL to mmol/L multiply by 0.0113.*

At follow-up (median=16.0 months; IQR = 12.0), median TSH was lower in treated patients (2.4 vs. 4.4 mIU/L, p=0.01). As well, median within-patient changes indicated decreases from baseline in both groups (ps<0.02), being greater in treated patients (-3.5 vs. -1.2 mIU/L, δ=0.41, p=0.03). Median lipid levels at follow-up did not statistically differ between groups. However, median within-patient changes in total cholesterol marginally differed by group (δ=0.36; p=0.05): trending upward in treated patients (+7.5 mg/dL or +0.2 mmol/L) and downward in untreated patients (-7.0 mg/dL or -0.2 mmol/L); neither change reached statistical significance. Additionally, median within-patient changes in LDL-cholesterol differed by group (δ=0.44; p=0.02): decreasing among untreated patients (-4.5 mg/dL or -0.2 mmol/L; p=0.03) and trending upward in treated patients (+12.5 mg/dL or +0.3 mmol/L) but not reaching statistical significance. HDL appeared generally stable over time for both groups. Triglycerides appeared to decrease in both treated (-13.5 mg/dL or -0.2 mmol/L) and untreated (-18.0 mg/dL or -0.2 mmol/L) patients, although, changes varied largely within patients and did not reach significance.

In **Table 2**, we summarize within and between group changes in hormonal and lipid profiles by follow-up, derived from statistical models that adjusted for baseline study covariates. Adjusted median TSH and lipid values at follow-up did not differ between groups. However, there were several findings regarding within-patient changes. Both treatment groups displayed decreases in median TSH by follow-up [treated=-2.5 mIU/L (95% CI: -3.8, -1.2), untreated=-1.7 mIU/L (-2.6, -0.8); ps<0.001] and did not differ between groups. Treated patients had increased median total cholesterol [+7.8 mg/dL (2.2, 13.5) or +0.2 mmol/L (0.1, 0.4); p=0.01] and LDL-cholesterol [+13.2 mg/dL (1.4, 25.1) or +0.3 mmol/L (0.04, 0.6); p=0.03] by follow-up, which was higher than untreated patients (δs=0.46 and 0.78; ps=0.005 and 0.001, respectively) who saw no substantial changes in total cholesterol [-5.1 mg/dL (-13.6, 3.4) or -0.1 mmol/L (-0.4, 0.1)] and LDL-cholesterol [-3.6 mg/dL (-10.9, 3.7) or -0.1 mmol/L (-0.3, 0.1)]. HDL and triglycerides did not change over time for either treatment group and did not differ between groups. Note, similar to the crude analyses, point estimates for triglycerides appeared to suggest decreases in both groups but changes again varied largely and did not reach statistical significance.

**Table 2:** Adjusted TSH and lipid metabolism changes from baseline.

	Treated			Untreated		
	Baseline	Adjusted follow-up	Adjusted change	Baseline	Adjusted follow-up	Adjusted change
Variable	Median (Q_1_, Q_3_)	Median (95% CI)	Median (95% CI)	Median (Q_1_, Q_3_)	Median (95% CI)	Median (95% CI)
TSH							
mIU/L	5.8 (5.3, 6.4)	3.3 (2.6, 4.2)	-2.5^***^ (-3.8, -1.2)	5.5 (5.1, 6.4)	3.8 (3.5, 4.4)	-1.7^***^ (-2.6, -0.8)
CHL							
mg/dL	197.0 (173.0, 218.8)	204.8 (180.8, 226.8)	7.8^**^ (2.2, 13.5)	207.5 (182.5, 226.0)	202.4 (182.9, 213.9)	-5.1 (-13.6, 3.4)
mmol/L	5.1 (4.5, 5.7)	5.3 (4.7, 5.9)	0.2^**^ (0.1, 0.4)	5.4 (4.9, 5.7)	5.2 (4.7, 5.5)	-0.1 (-0.4, 0.1)
LDL							
mg/dL	110.0 (100.0, 127.0)	123.2 (98.2, 151.2)	13.2^*^ (1.4, 25.1)	121.0 (102.5, 133.5)	117.4 (107.4, 128.4)	-3.6 (-10.9, 3.7)
mmol/L	2.8 (2.2, 3.6)	3.2 (2.5, 3.9)	0.3^*^ (0.04, 0.6)	3.1 (2.9, 3.4)	3.0 (2.8, 3.3)	-0.1 (-0.3, 0.1)
HDL							
mg/dL	48.0 (43.2, 51.5)	50.7 (45.7, 54.7)	2.7 (-4.3, 9.8)	53.0 (43.5, 59.0)	54.2 (49.2, 57.2)	1.2 (-2.0, 4.5)
mmol/L	1.2 (1.1, 1.3)	1.3 (1.2, 1.4)	0.1 (-0.1, 0.3)	1.4 (1.2, 1.5)	1.4 (1.3, 1.5)	0.0 (-0.1, 0.1)
Triglycerides							
mg/dL	112.5 (91.5, 144.2)	106.1 (83.6, 147.6)	-6.4 (-46.8, 34.0)	116.0 (85.0, 150.0)	97.9 (92.9, 118.9)	-18.1 (-42.8, 6.5)
mmol/L	1.3 (1.0, 1.7)	1.2 (0.9, 1.7)	-0.1 (-0.5, 0.4)	1.3 (1.3, 1.5)	1.1 (1.0, 1.3)	-0.2 (-0.5, 0.1)

*p<0.05. **p<0.01. ***p<0.001. TSH, Thyroid-stimulating hormone. CHL, Total cholesterol. LDL, LDL cholesterol. HDL, HDL cholesterol.

## Discussion

Based on our study results, patients with mild SH who were treated with LT4 were significantly more likely to experience an increase in total and LDL-cholesterol by follow-up, higher than untreated patients. Not surprisingly, the TSH of untreated patients was reduced on repeated observations (16). The increase in total and LDL-cholesterol in the treated group is different from the findings of a previous meta-analysis which identified a lipid lowering effect of LT4 on total and LDL-cholesterol across 12 randomized placebo controlled trials with a total of 940 patients with SH, defined by a normal free T4 and elevated TSH value (10). The mean baseline TSH value across all the studies was 6.8 mIU/L, and mean baseline values ranged from 5.3-11.7 mIU/L. The lipid lowering effect was greatest in patients who received less than 6 months of treatment. It is difficult to directly compare the studies because of differences in baseline TSH, LT4 dosage, and length of time between baseline and final TSH and lipid values. Additionally, only 4 of the 12 studies were limited to patients with mild TSH (defined as a baseline TSH <10 mIU/L) (17–20). Of the 4 studies, only 2 mentioned excluding patients treated with statins (17, 20); both studies reported a decrease in lipids following LT4 therapy. Interestingly, in one of these studies a decrease in lipids was also observed in the control group (20). Of note, 6 of the 12 studies included a treatment duration of less than 12 months, and only one included a treatment duration of more than 12 months (20). The average length of treatment in our retrospective analysis was 16 months.

In hypothyroidism, monotherapy with LT4 has been shown to normalize TSH, but results in a shift toward an increase in free T4 and reduction in T3 levels (12). In a recent interventional study in patients undergoing total thyroidectomy we observed that compared to pre-surgical euthyroidism, once treated with LT4, patients experienced an increased in cholesterol despite normalization of the TSH levels. Conversely, free T4 levels increased while total T3 levels decreased (21). This is consistent with the hypothesis that LT4 alone is not sufficient in restoring euthyroidism in all targets of thyroid hormone action. In the context of mild SH LT4 therapy could result in suppression of the residual endogenous production of thyroid hormone, particularly T3, leading to a paradoxical decrease in thyroid hormone action. Our results appear to support this hypothesis. To this end, lipid metabolism is exquisitely sensitive to thyroid hormone action and it is, at the individual basis, a highly sensitive index of thyroid hormone action (22, 23).

Our study has several limitations, including the inherent shortcomings of a retrospective analysis, the small sample size, and the lack of measurement of free T4 levels beyond baseline. T3 levels were not recorded as well. Additional limitations include the inability of assessing the use of nutraceuticals (24) or substances (*e.g.* biotin) which can affect thyroid hormone assays. On the other hand, strengths of this study include the selection of a study population affected by mild SH and devoid of confounder effect of lipid lowering therapy and medications which affect thyroid hormone homeostasis. This in turn provided the opportunity to evaluate the association of LT4 therapy and changes in lipid metabolism parameters.

## Conclusions

In this retrospective study, we uncovered an unexpected counterintuitive relation between LT4 treatment and lipid metabolism in patients affected by mild SH which challenges current clinical recommendations. Further observational research in large datasets as well interventional studies are necessary to determine if long-term LT4 treatment is beneficial in the treatment of mild SH, or if it could paradoxically increase cholesterol levels, and potentially worsen cardiovascular risk.

## Data Availability

The raw data supporting the conclusions of this article will be made available by the authors, without undue reservation.
